# Onymity promotes cooperation in social dilemma experiments

**DOI:** 10.1126/sciadv.1601444

**Published:** 2017-03-29

**Authors:** Zhen Wang, Marko Jusup, Rui-Wu Wang, Lei Shi, Yoh Iwasa, Yamir Moreno, Jürgen Kurths

**Affiliations:** 1 Center for OPTical IMagery Analysis and Learning (OPTIMAL), Northwestern Polytechnical University, Xi’an 710072, China.; 2 Interdisciplinary Graduate School of Engineering Sciences, Kyushu University, Fukuoka 816-8580, Japan.; 3 Research Center of Mathematics for Social Creativity, Hokkaido University, Sapporo 060-0812, Japan.; 4 Center for Ecology and Environmental Sciences, Northwestern Polytechnical University, Xi’an 710072, China.; 5 State Key Laboratory of Genetic Resources and Evolution, Kunming Institute of Zoology, Chinese Academy of Sciences, Kunming 650223, China.; 6 School of Statistics and Mathematics, Yunnan University of Finance and Economics, Kunming 650221, China.; 7 Faculty of Science, Kyushu University, Fukuoka 819-0395, Japan.; 8 Institute for Biocomputation and Physics of Complex Systems (BIFI), University of Zaragoza, 50009 Zaragoza, Spain.; 9 Department of Theoretical Physics, University of Zaragoza, 50009 Zaragoza, Spain.; 10 Institute for Scientific Interchange (ISI), ISI Foundation, 10126 Turin, Italy.; 11 Potsdam Institute for Climate Impact Research (PIK), 14473 Potsdam, Germany.; 12 Department of Physics, Humboldt University, 12489 Berlin, Germany.; 13 Institute for Complex Systems and Mathematical Biology, University of Aberdeen, Aberdeen AB24 3UE, U.K.

**Keywords:** Evolutionary Game Theory, human behavior, Prisoner’s Dilemma, defection, punishment, reciprocity

## Abstract

One of the most elusive scientific challenges for over 150 years has been to explain why cooperation survives despite being a seemingly inferior strategy from an evolutionary point of view. Over the years, various theoretical scenarios aimed at solving the evolutionary puzzle of cooperation have been proposed, eventually identifying several cooperation-promoting mechanisms: kin selection, direct reciprocity, indirect reciprocity, network reciprocity, and group selection. We report the results of repeated Prisoner’s Dilemma experiments with anonymous and onymous pairwise interactions among individuals. We find that onymity significantly increases the frequency of cooperation and the median payoff per round relative to anonymity. Furthermore, we also show that the correlation between players’ ranks and the usage of strategies (cooperation, defection, or punishment) underwent a fundamental shift, whereby more prosocial actions are rewarded with a better ranking under onymity. Our findings prove that reducing anonymity is a valid promoter of cooperation, leading to higher payoffs for cooperators and thus suppressing an incentive—anonymity—that would ultimately favor defection.

## Teaser

Researchers test whether and how onymity, as opposed to anonymity, promotes cooperation in a social dilemma experiment.

## INTRODUCTION

Why is cooperation so prevalent in nature? After all, Darwinian selection should result in individuals pursuing their own selfish interests (*1*). However, cooperation not only prevails, but its mass emergence is the main force behind the evolutionary transitions from single-cell organisms to complex animal and human societies (*2*). To systematically analyze the fundamental trade-off faced by individuals who choose between cooperating and free-riding behaviors, mathematicians M. M. Flood and M. Dresher devised in the 1950s a model of conflict and cooperation known as Prisoner’s Dilemma (PD) (*3*). In each realization of PD, two players must select between cooperation (*C*) and defection (*D*). Although mutual cooperation generates a higher collective payoff, both players are tempted to defect because they can do better individually by exploiting a cooperative opponent. Without any external help, natural selection favors mutual defection (*4*).

Attempts to offset the unfavorable evolutionary outcome of PD branched into two complementary lines of research. One was spurred by the result that in spatially structured populations, cooperators who aggregate into clusters may avoid being wiped out by defectors (*5*, *6*). Subsequent interest in the role of spatial topology in this field led to the introduction of complex networked structures (*7*–*9*) and, more recently, to a number of experiments examining the evolution of cooperation in relation to network heterogeneity, dynamics, and updating rules (*10*–*13*). The other line of research focused on social mechanisms, whereby individuals rely on common knowledge about their opponents or otherwise actively try to reduce the potential benefit of free riders. Examples of these mechanisms include tit for tat (*14*), win-stay, lose-shift (*15*), reputation (*16*), reward (*17*), and costly punishment (*18*–*25*). The special attention devoted to costly punishment (*P*) as an independent strategy partly originated from the controversial readiness of punishers to incur a cost to make other individuals pay an even higher fine. The role of this strategy in the promotion of cooperation has been disputed, with particularly negative views expressed on the basis of social experiments (*22*, *24*).

Much of the mentioned progress in understanding the evolution of cooperation has been ascribed to several mechanisms that promote cooperative behavior (*26*). Although the details of these mechanisms differ greatly, they have a common basis (*27*–*30*) interpretable as the information needed to reduce anonymity relative to a primitive, well-mixed population (*31*, *32*). Therefore, in the context of theoretical hypotheses and experimental evidence (*30*, *33*), a question of considerable interest arises: Would reducing the anonymity of opponents in an actual social experiment promote cooperation? We set to answer this question by investigating the differences between anonymous and onymous treatments in a repeated PD experiment. We consider extended PD with three strategies [cooperation (*C*), defection (*D*), and punishment (*P*)]—as in the study of Dreber *et al.* (*22*) and in the study of Wu *et al.* (*24*)—to further inspect the (controversial) effects of costly punishment and to obtain richer correlations between payoffs and the strategies used, as compared to what would be possible with traditional two-strategy PD.

A total of 154 undergraduates voluntarily participated in a repeated PD experiment at Yunnan University of Finance and Economics (for more details, see Materials and Methods and the Supplementary Materials). The experiment consisted of pairwise encounters under two treatments: anonymous (T1) and onymous (T2). In T1, each participant interacted anonymously with opponents, whereas in T2, opponents were made known. In either of the treatments, the participants did not know how many rounds a particular interaction would have, but they knew that the probability of entering the next round had been set to 75%. When an interaction ended, the next set of previously unrealized pairwise encounters was drawn randomly until all participants interacted with each other. Participants were instructed to choose one action in each round, where actions reflected the three possible strategies (*C*, *D*, or *P*). The outcomes of unilateral actions were defined such that cooperation (*C*) meant paying 1 unit for the opponent to receive 2 units, defection (*D*) meant gaining 1 unit at a cost of 1 unit for the opponent, and punishment (*P*) meant paying 1 unit for the opponent to lose 4 units. The resulting unilateral and bilateral payoff matrices are given in Eq. 1

$$\begin{array}{c} \begin{array}{cccccc} \text{Own} & \text{Foe’s} & & C & D & P \end{array}\\ \begin{array}{c} C\\ D\\ P \end{array}[\begin{array}{cc} -1 & 2\\ 1 & -1\\ -1 & -4 \end{array}]\Rightarrow [\begin{array}{ccc} 1 & -2 & -5\\ 3 & 0 & -3\\ 1 & -2 & -5 \end{array}] \end{array}$$ (1)

## RESULTS

**Fig. 1:**
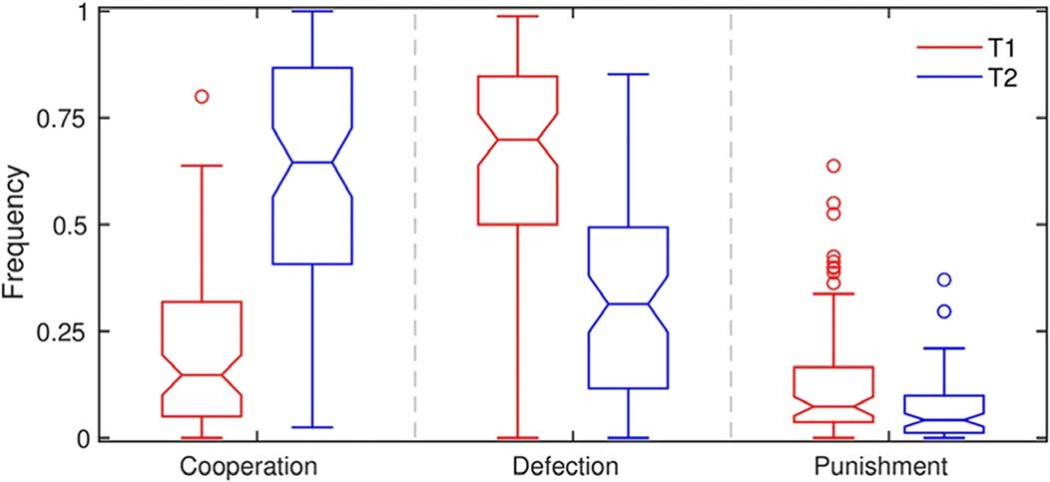
Onymity brings out the best in people. Cooperation takes over defection as the dominant action if the anonymous treatment (T1) is replaced with the onymous one (T2). Pairwise comparisons indicate that the increase in the frequency of cooperation and the decrease in the frequency of defection between the two treatments are significant at the 5% level. The frequency of punishment decreases, but the difference between the medians is insignificant. Box-and-whisker plots with notches reveal the empirical distribution of the frequency of each action. Box height indicates the interquartile range with the median in between. Notches indicate the 95% confidence intervals for the median, thus permitting a visual pairwise comparison. Whisker height is such that 99.3% of the normally distributed data would be covered. The points drawn as outliers fall outside of the whisker coverage.

A comparison of the results from the two experimental treatments reveals emerging prosocial behavior under onymity (Fig. 1). We use the term prosocial in the sense of benefiting the collective. Cooperation is thus a prosocial action because if players in PD cooperate, then the group payoff is maximized. Conversely, defection is an antisocial action because defectors profit from exploiting cooperators, which in turn decreases the group payoff. Costly (altruistic) punishment is somewhat ambiguous because its immediate effect on the payoff is negative. However, a punisher’s presumable intention is to force a defector into a more cooperative frame of mind and, consequently, to generate a positive indirect effect on the group payoff. The empirical findings do not seem to confirm this indirect effect of punishment (*22*, *24*)—an issue to which we devote considerable attention here. We find that the behavior of the participants differs significantly between anonymous and onymous treatments (χ^2^ test, χ^2^ = 2233, *P* < 10^−6^). Pairwise comparisons (Fig. 1) show an increase in the frequency of cooperation and a decrease in the frequency of defection, both significant at the 5% level, if anonymity is replaced with onymity. The frequency of punishment also notably decreases, but the difference in the medians is not significant. Overall, a more prosocial behavior emerges under onymity. This conclusion is consistent with the theoretical findings, whereby cooperation-promoting mechanisms—all of which preclude complete anonymity—can sometimes change the nature of PD (selection favors defection) to the point of eliminating the dilemma altogether (selection favors cooperation) (*31*).

**Fig. 2:**
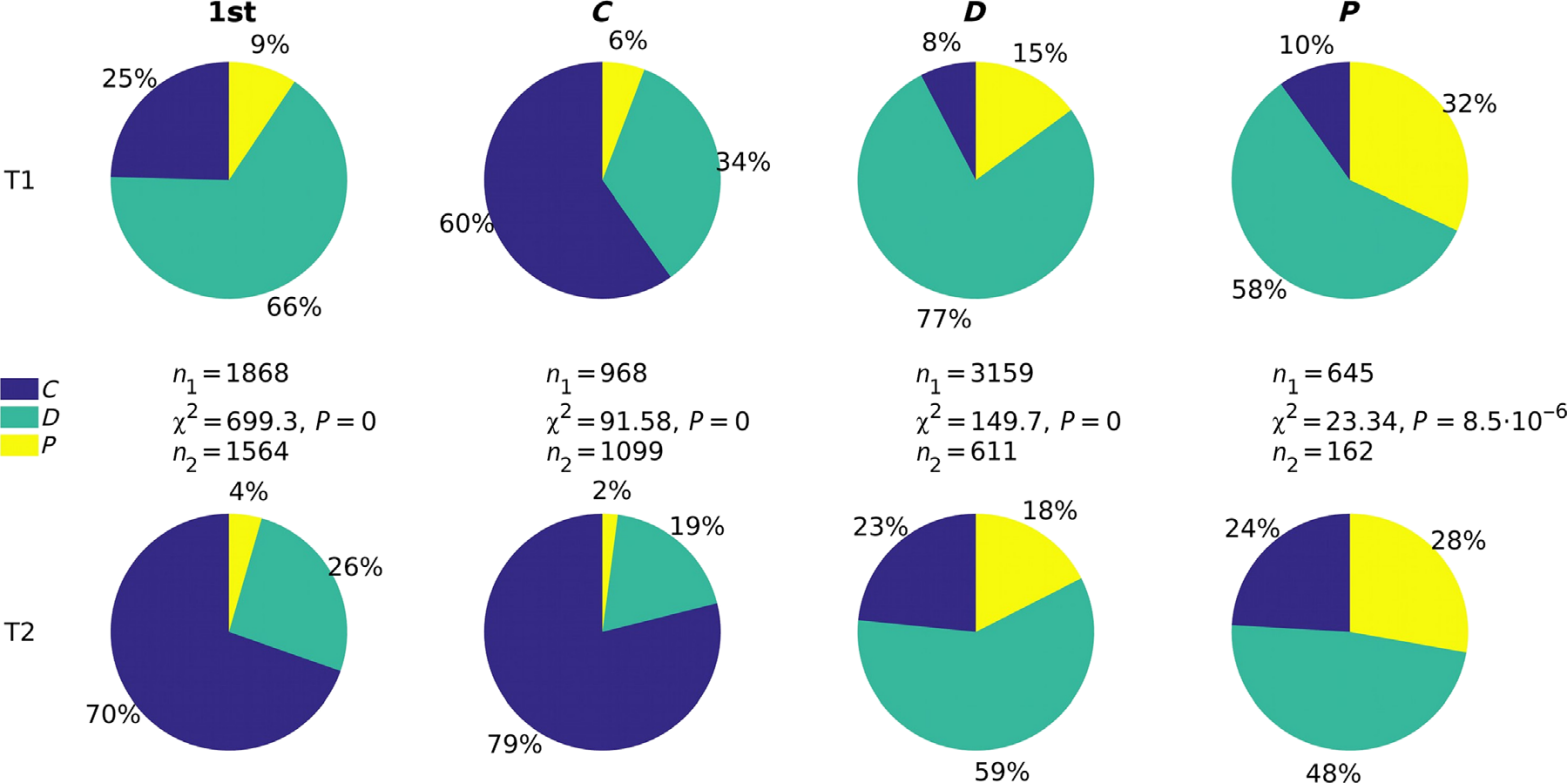
Onymity elicits prosocial behavior, but punishment does not. The action in the first round (column 1st) and the first-order conditional strategies (columns *C*, *D*, and *P*, indicating the responses to cooperation, defection, and punishment, respectively) in later rounds are all significantly different between the two experimental treatments. The increase in cooperation and the decrease in defection by switching from the anonymous (T1) to the onymous (T2) treatment is particularly noticeable. Thus, onymity elicits prosocial behavior. In contrast, an immediate response to punishment—the preferred choice being defection followed by counter-punishment—is overwhelmingly antisocial even under onymity.

A closer look into participants’ behavior reveals that the action chosen in the first-round and the first-order conditional strategies (Fig. 2) becomes more prosocial under onymity (T2) compared to anonymity (T1). The increase in the frequency of cooperation and the decrease in the frequency of defection is particularly noticeable. In contrast, the response to punishment is decisively antisocial, with defection being the preferred choice followed by counter-punishment. In T1, punishment was countered with cooperation in less than 10% of cases. Although the situation improved in T2, cooperation still remained the least-preferred option. Hence, even if the intention of punishers is ultimately prosocial, the immediate result of their action is not. Another interesting outcome in T1 is that punishment was chosen as the first-round action about 100% more often than in the study of Dreber *et al.* (*22*), which is qualitatively similar to, but not as marked as, the result from the study of Wu *et al.* (*24*). The large difference between the choices in the two cited studies was attributed to the different cultural backgrounds of participants (Boston, MA and Beijing, China, respectively) (*24*). The same explanation may apply to our result relative to the study of Dreber *et al.* (*22*), given that the present experiment was also conducted in China. Among Chinese students, the frequency of punishment in the first round dropped below 5%—the level recorded in the Boston experiment—only after onymity had been introduced (that is, in T2). For a more detailed comparison between the present study and the study of Dreber *et al.* (*22*) and the study of Wu *et al.* (*24*), including the implications for the role of punishment in increasing the degree of cooperativeness in social dilemmas, see the Supplementary Materials.

**Fig. 3:**
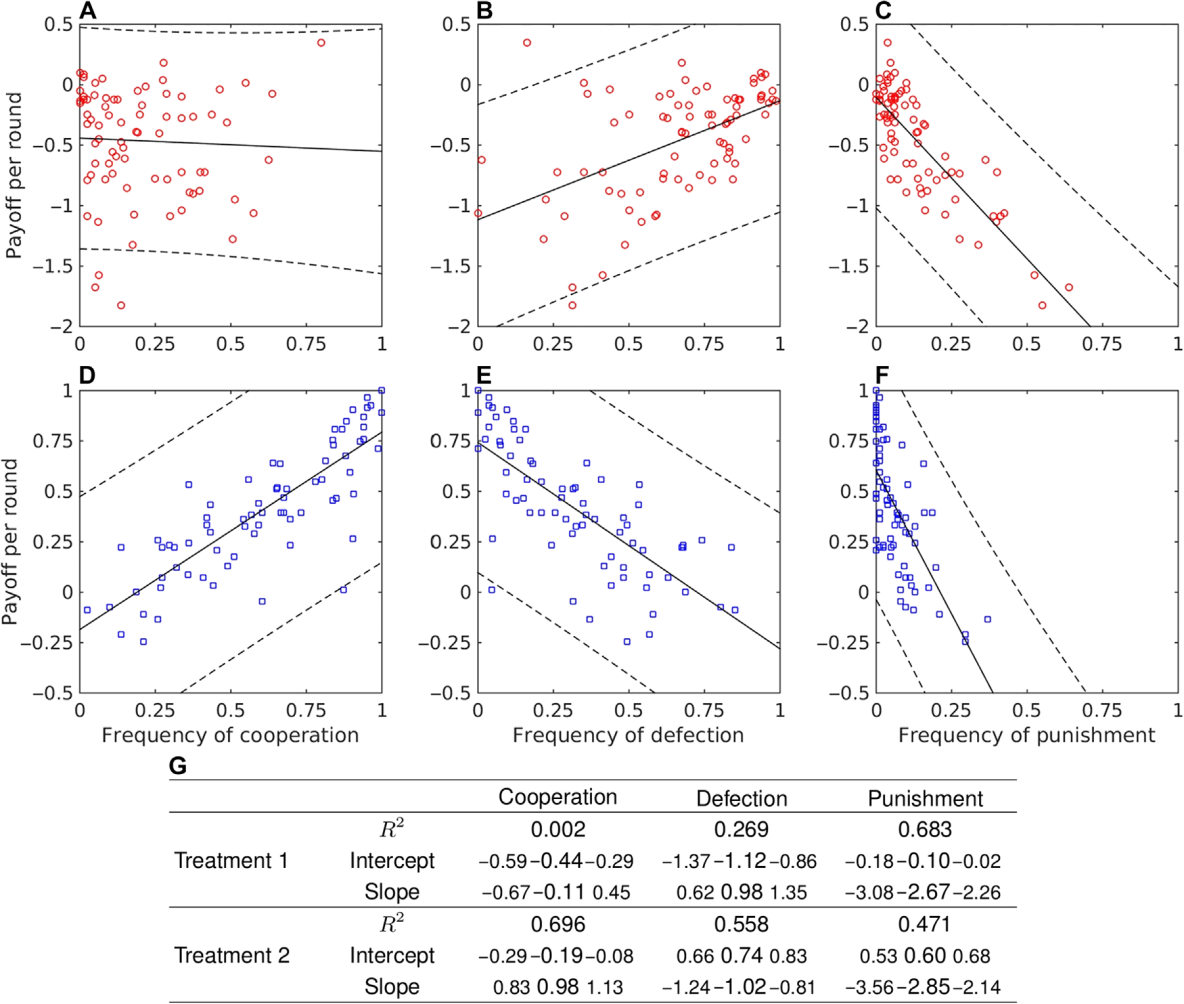
Playing nice under onymity pays off. (**A** and **B**) When the opponent remains unknown (T1), the payoff per round does not correlate with the use of cooperation but correlates positively with the use of defection [in contrast with the study of Dreber *et al.* (*22*)], thus indicating that the prosocial action (that is, cooperation) is less desirable than the antisocial one (that is, defection). (**D** and **E**) When the opponent is known (T2), the payoff per round correlates positively with the use of cooperation and negatively with the use of defection, showing that the prosocial action is now more desirable than the antisocial one. (**C** and **F**) The only similarity between the two treatments is that the payoff per round correlates negatively with the use of punishment [the “winners don’t punish” effect (*22*, *24*)]. Shown are the regression lines with the 95% prediction intervals (dashed curves). (**G**) In accompanying statistical analysis, the smaller font size indicates the 95% statistical confidence intervals.

**Fig. 4:**
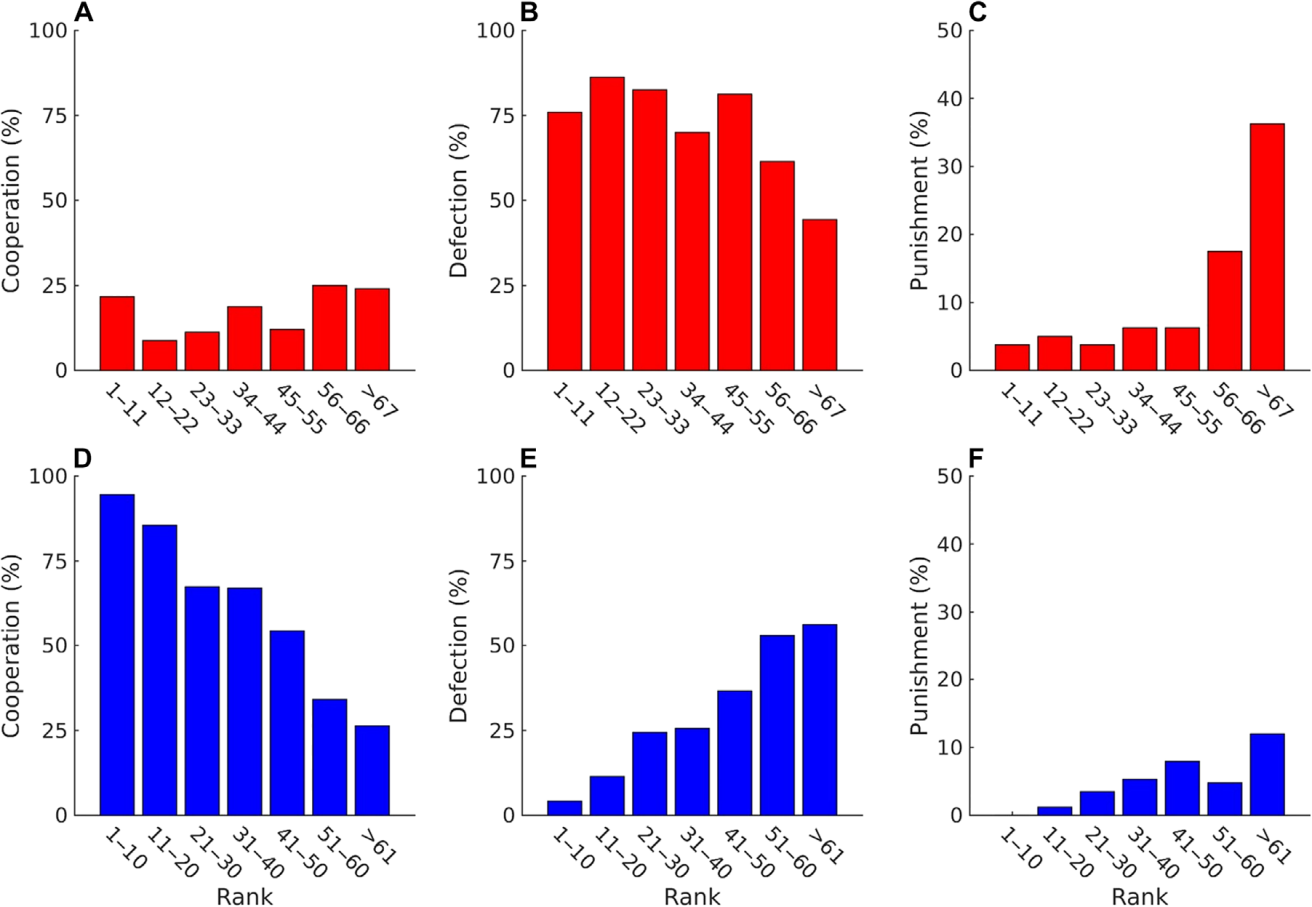
Onymity alters the factors of success in a social dilemma. (**A** and **B**) Under anonymity (T1), the use of cooperation does not affect the ranking, whereas the use of defection is advantageous, albeit in a limited fashion. (**D** and **E**) Under onymity (T2), the use of cooperation is decisively advantageous for the ranking of the participants. In contrast, the use of defection is disadvantageous. (**C** and **F**) The “winners don’t punish” effect remains unaltered between the two experimental treatments. The standard competition ranking is used, such that a lower ranking number is better. The vertical axes report the average use of each action per 100 rounds. For clarity, the scale for the use of punishment is doubled. The number of bins in the histograms is kept the same for both treatments, although the number of participants was slightly higher in T1 than in T2 (80 versus 74, respectively), thus causing the bin sizes to slightly differ between the treatments.

Because of the considerable changes in the behavior of participants between the two experimental treatments (Figs. 1 and 2), one could also anticipate major differences in the payoff per round. The median payoff per round of −0.319 under anonymity (T1) is significantly lower than the corresponding value of 0.395 under onymity (T2; Wilcoxon rank sum test, *W* = 8491, *P* < 10^−6^). Furthermore, the relationship between the choice of action and the performance of participants in terms of the payoff per round reveals contrasting outcomes between the two experimental treatments (Fig. 3). In T1, the payoff per round is uncorrelated with the frequency of cooperation (*F* test, *F* = 0.151, *P* = 0.698; however, see regression diagnostics in the Supplementary Materials), but it is positively correlated with the frequency of defection (*F* = 28.7, *P* < 10^−6^; Fig. 3, A and B). The latter result, although in contrast with the study of Dreber *et al.* (*22*), is not entirely surprising because in PD, selection favors defection. Given a sufficient number of cooperators to exploit, defectors necessarily attain a relative advantage in payoff. A conclusion is that, under anonymity, the antisocial action may produce a more desirable outcome in terms of the payoff per round than the prosocial one. However, in T2, the situation is reversed. The payoff per round becomes positively correlated with the frequency of cooperation (*F* = 165, *P* < 10^−6^) and negatively correlated with the frequency of defection (*F* = 91.0, *P* < 10^−6^; Fig. 3, D and E). Thus, onymity elicits prosocial behavior among participants, one that also pays back—winners play nice (Fig. 4).

**Fig. 5:**
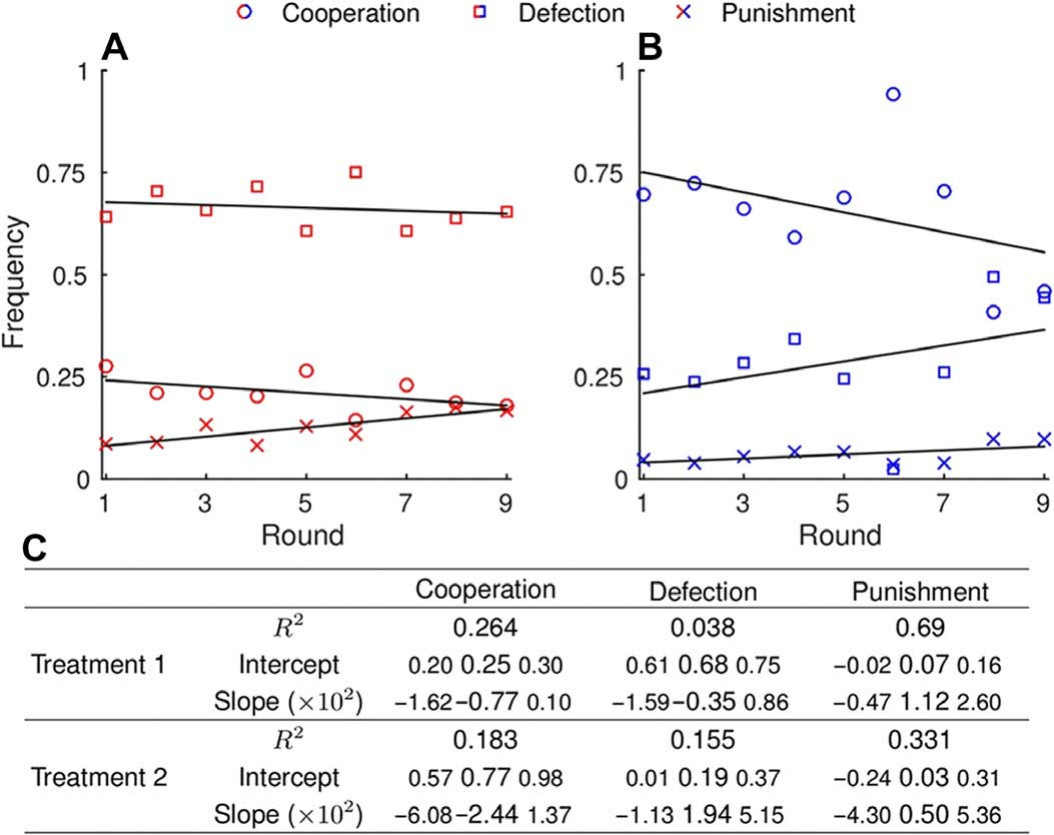
Frequency of cooperation, defection, and punishment over the course of an interaction. (**A**) In the anonymous treatment (T1), action frequencies do not show a statistically discernible trend as the interaction progresses, thus contrasting the results in the study of Dreber *et al.* (*22*) and in the study of Wu *et al.* (*24*). (**B**) In the onymous treatment (T2), the frequency of cooperation (defection) seems to be decreasing (increasing), but again, the results are statistically insignificant. Shown are the data points (circles, squares, and x marks for cooperation, defection, and punishment, respectively) and the regression lines. The latter were simultaneously fitted to ensure that the frequencies add up to unity. (**C**) In the accompanying statistical analysis, the smaller font size indicates the 95% statistical confidence intervals. We additionally tested whether the angle between the regression lines for cooperation and defection was significantly different from zero, but we obtained a negative result (*P* = 0.58 and *P* = 0.072 for T1 and T2, respectively).

The consequences of the use of punishment on the payoff per round further corroborate the conclusion that winners play nice. We find that the payoff per round is negatively correlated with the frequency of punishment under anonymity (*F* = 168, *P* < 10^−6^) and even more so under onymity (*F* = 64.1, *P* < 10^−6^; Fig. 3, C and F). These results are a manifestation of the “winners don’t punish” effect observed in previous studies (*22*, *24*). What is truly remarkable about this effect is its reproducibility even in the onymous treatment, which is, as we have seen, sufficient to drastically alter the outcomes of other actions (Fig. 4). The issue of reproducibility is an important one because not all outcomes of previous experimental studies are readily obtained. For instance, the frequency of punishment in the first round under anonymity, although double that of the study of Dreber *et al.* (*22*), is still 50% lower than the frequency found in the study of Wu *et al.* (*24*). This difference is quantitatively large, suggesting that the cultural background used to explain this result in the first place may be a dominant, but not the only, factor affecting the behavior of participants. In general, confounding factors other than cultural background may have affected our results. We had sufficient data to analyze two of these factors, gender and academic background. The results of these analyses are outlined in the Supplementary Materials. Another example of a result that differs from previous studies is our failure to observe the increasing frequency of defection with the number of rounds played (Fig. 5) (*22*, *24*). The reason may again be the lack of a unique underlying mechanism—the study of Dreber *et al.* (*22*), for example, offers two possible explanations as to why the frequency of defection could increase with the number of rounds. The described mismatches between the experiments highlight the importance of understanding the mechanisms behind the statistically significant results. Presumably, the simpler and more stable these mechanisms are, the more likely the result is to be reproducible.

**Fig. 6:**
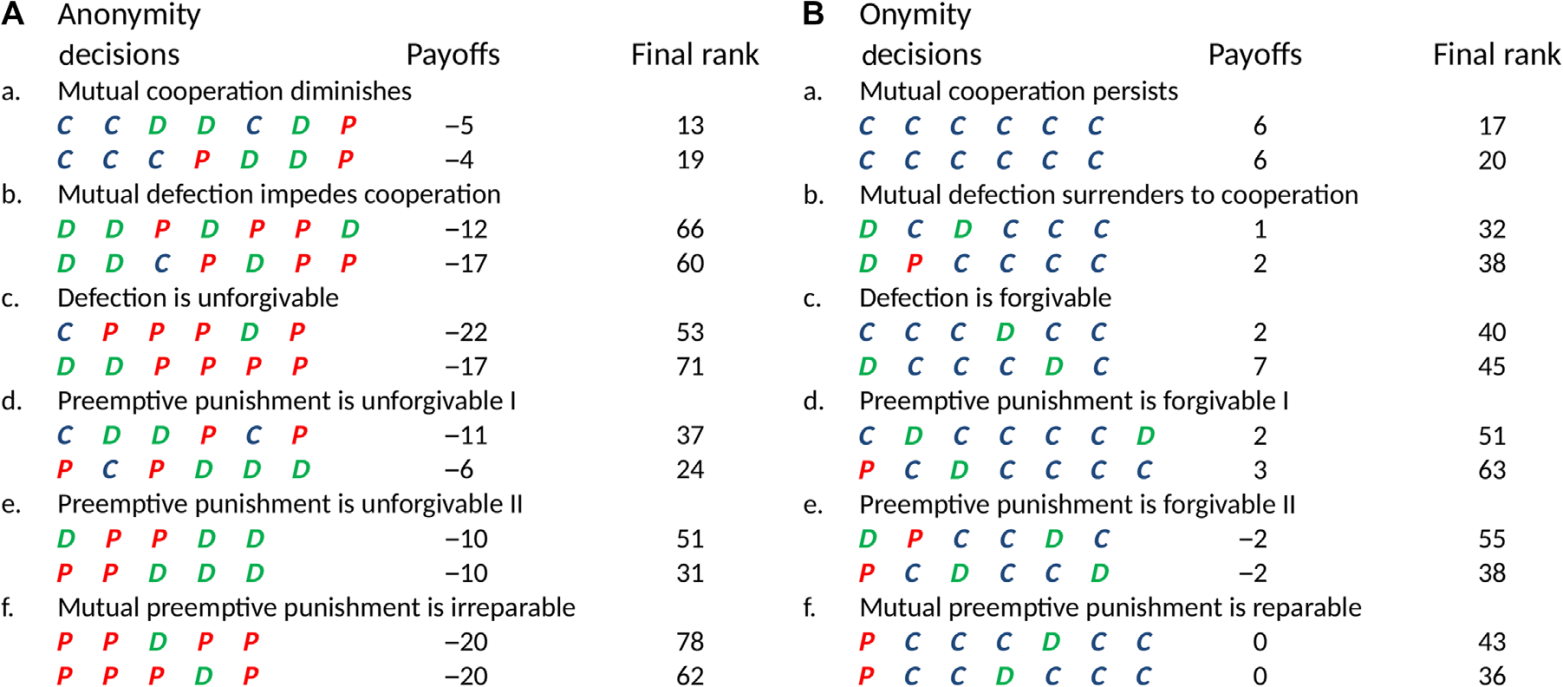
Same beginnings but vastly different endings. Inner workings of anonymity and onymity through a direct comparison of some typical interactions that finished differently although they started the same. (**A**) In the anonymous treatment, even mutual cooperation occasionally gives way to defection and punishment. Other initial decisions make it only harder to reach and maintain a cooperative outcome. (**B**) In the onymous treatment, in contrast, even mutual preemptive punishment is reparable. Other initial decisions make it mostly easier to reach and maintain a cooperative outcome.

We took two preliminary steps toward a more mechanistic understanding of the observed empirical patterns. First, we peeked into the inner workings of anonymity and onymity by comparing typical interactions that, although they had started the same, ended up very differently depending on the treatment (Fig. 6). In the anonymous treatment (T1), even those interactions that had begun with mutual cooperation, more often than not, spiraled out of control in a series of antisocial decisions (namely, mutual defections or retaliatory punishments). Establishing and maintaining a cooperative outcome in instances in which the first round had consisted of decisions other than mutual cooperation proved all the more difficult. These results are in stark contrast with the typical interactions in the onymous treatment (T2). Under onymity, the relationship between two opponents was often reparable even after opening the interaction with mutual punishment. All other first-round decisions mostly led to establishing and maintaining a cooperative outcome, provided that the interaction lasted for a sufficient number of rounds.

As a second step toward a more mechanistic understanding of the empirical patterns, we recreated the experiment using computer simulations (see the Supplementary Materials for details). By making three relatively straightforward assumptions, we could qualitatively and even quantitatively reproduce the experimental results and thus obtain some rudimentary indications of how to understand behaviorisms prompted by a social dilemma. Specifically, first-order conditional strategies seem to be followed on average, determining in the process whether prosocial tendencies will be rewarded or not. There is a considerable variability among individuals. However, overall behavior resembles the selection of strategies by trial and error, the main purpose of which seems to be filtering out maladjusted strategies rather than actively seeking well-adjusted ones.

## DISCUSSION

In conclusion, onymity entices cooperation. To solidify this conclusion, we performed two additional control trials, one under anonymous and the other under onymous conditions (see the Supplementary Materials for details), wherein participants played a more traditional 2 × 2 PD game without punishment. Qualitatively similar results were obtained in these control trials, thus more firmly establishing the position of onymity as a powerful promoter of cooperation. Further quantitative comparisons between the controls and treatments T1 and T2 indicated that punishment, at best, failed in increasing the degree of cooperativeness among participants. However, an even more important result is that the level of cooperation may be sufficient to make onymous prosocial behavior advantageous—winners play nice. The conclusion that winners play nice under onymity is a major step forward from the previous studies in, at least, a twofold manner. The study of Dreber *et al.* (*22*), for example, establishes that successful individuals in an unfavorably structured social dilemma (of which PD is a prime example) avoid punishing others. This finding is complemented by our results because when the cloak of anonymity is removed, successful individuals should aspire to more than just staying shy of punishing others—they should behave truly prosocially and cooperate in the face of a strong temptation to defect. Furthermore, Wu *et al.* (*24*) report how costly punishment fails to increase cooperation. Although this result is interesting in itself, we actually offer a potential remedy for situations in which punishment has failed. Overall, the results are supportive of a notion that for cooperative behavior to emerge, opponents need some information about each other. This notion may have major implications for theoretical studies seeking mechanisms that promote cooperation. Instead of scouring an endless landscape of possible scenarios, the focus may shift toward available information for mutual recognition (*27*–*30*). At present, for instance, what degree of onymity effectively supports cooperation remains an interesting unknown.

Throughout the experimental sessions, we operationalized onymity simply by allowing opponents to learn each other’s name. However, for this to be effective, we relied on the fact that participants were students who had attended the same classes before. Thus, our sample exhibited two important characteristics that suggest a rather high level of onymity. One characteristic is that students had previous knowledge about one another, and the other is homogeneity in terms of age and interests, making mutual recognition and association relatively easy. This high level of onymity bears the question: To what extent could onymity be lowered and still promote cooperation? Although an answer is beyond the scope of the present study, we envisioned setups that would allow rigorous experimentation in this direction. For example, recruits need not be acquaintances before the experiment (nor do they have to originate from the same social groups) yet might be required to introduce themselves to others and even spend some time having a conversation about a given topic. This experimental design would certainly preclude complete anonymity, but neither mutual recognition nor association would be as easy as in the experiments presented here.

As with any experimental study, there is a danger in extrapolating the results outside the narrow set of controlled conditions and parameters prevalent during the experiment. We already highlighted several important differences between this and previous comparable studies, thereby hinting at plausible behavioral determinants operating in the face of a social dilemma (for example, cultural background). Aside from the cultural background, a slew of economic (for example, income and wealth) and biological (for example, age and gender) parameters may also play an important role, begging the question how robust the results of experimental studies truly are. It is somewhat encouraging in this context that computer simulations built on a small set of straightforward assumptions successfully reproduce the experimental results. These simulations justify our focus on the first-order conditional strategies in interpreting the raw data, but they fall short of explaining what overrides are triggered by onymity in order for defection in T1 to be replaced with cooperation in T2. At present, we can only speculate that participants anticipate to be reciprocated for prosocial actions under onymity despite being unable to spread rumors or to interact more than once (a few PD rounds) with any given individual.

## MATERIALS AND METHODS

### Experimental methods

We gathered 154 voluntary undergraduate students (49.4% women and 50.6% men, mean age of 21.6; for more details, see the Supplementary Materials) from three universities in Kunming, China, who major in mathematics (55 people), statistics (39 people), and eight other, mostly social sciences and humanities (60 people). Participants engaged in a repeated PD experiment at the computer laboratory of Statistics and Mathematics School, Yunnan University of Finance and Economics, consisting of around 100 isolated computer cubicles running z-Tree (*34*). A cubicle was randomly assigned to each participant, who would then read instructions about experimental procedures and have the understanding thereof tested via a questionnaire (fig. S1). Any two participants were separated by a vacant cubicle to help two supervisors, who could answer technical questions too, maintain order.

The experiment consisted of six sessions—three per treatment—which took place on 6 September, 11 October, and 15 November 2014 for the anonymous (T1; 26.7 participants per session) and on 20 September, 25 October, and 8 November 2014 for the onymous treatment (T2; 24.7). Each session began with a practice interaction, followed by a series of interactions in which every participant interacted with everyone else in a random order. An interaction meant that a pair of opponents repeatedly faced the same PD, where the next round would occur with 75% probability. Participants in both treatments had half a minute to inspect the result of the preceding round and their total payoff. Furthermore, in T2, participants—strictly classmates—could see each other’s name. Sessions lasted approximately 1.25 hours. No participant engaged in multiple sessions.

As an incentive, in addition to a ¥15 show-up fee, all participants started with an initial score of 50 points, and it was explained that their final score, if positive, would be exchanged for a real currency at the rate of ¥0.2 per point. This setup generated the average earnings of ¥18.5, ranging from ¥15.0 to ¥31.6.

### Statistical methods and analyses

To get a sense of how the use of strategies (*C*, *D*, or *P*) is distributed without referring to a model distribution (for example, the normal distribution), we resorted to nonparametric descriptive statistics, that is, the median and the interquartile range. Subsequently, a direct comparison of the differences in the use of strategies between the two treatments (T1 and T2) was made on the basis of the median’s 95% confidence intervals. The first-order conditional strategies were naturally represented by means of 3 × 2 contingency tables, where table rows (columns) corresponded to *C*, *D*, and *P* (T1 and T2). Whether the strategies are independent of the treatments was tested with χ^2^ test. Using this test was justified by a large number of data points at our disposal.

The correlation between the payoff per round and the use of strategies was analyzed with ordinary least-squares regression and outlier detection methods, as presented in the study of Chatterjee and Hadi (*35*). Outlier detection was performed in conjunction with the tests of normality for residuals to identify instances in which a few data points had the potential to overwhelmingly affect the statistical inference. In contrast, in correlating the use of strategies with the number of rounds played, we used a custom-built regression routine for two reasons. First, the sum of the three strategies always equaled unity, implying a constraint that needed to be taken into account. Second, we used resampling (that is, bootstrapping) of the residuals because no indication of their true distribution was available from a low number of data points.

## Supplementary Material

http://advances.sciencemag.org/cgi/content/full/3/3/e1601444/DC1

## SUPPLEMENTARY MATERIALS

Supplementary material for this article is available at http://advances.sciencemag.org/cgi/content/full/3/3/e1601444/DC1 Supplementary Materials and Methods Supplementary Results fig. S1. Snapshot of the questionnaire used to test the basic understanding of PD games. fig. S2. Interface for playing the PD game in anonymous treatment. fig. S3. Interface for playing the PD game in onymous treatment. fig. S4. Control trials. fig. S5. Regression diagnostics. fig. S6. Computer-simulated recreations of the anonymous treatment (T1). fig. S7. Computer-simulated recreations of the onymous treatment (T2). fig. S8. Comparison with other similar studies. table S1. Basic information on the experimental sessions. table S2. Gender as a confounding factor. table S3. Academic background as a confounding factor. Reference (*36*)
