# Supplementary material for: Onymity promotes cooperation in social dilemma experiments
Source: Sci Adv. 2017 Mar 29;3(3):e1601444. doi: 10.1126/sciadv.1601444 (PMC5371422; doi:10.1126/sciadv.1601444)
Supplement: http://advances.sciencemag.org/cgi/content/full/3/3/e1601444/DC1 [file supp_3_3_e1601444__index.html]

Science Advances | Science Advances

## Supplementary Materials

**This PDF file includes:**

- Supplementary Materials and Methods
- Supplementary Results
- fig. S1. Snapshot of the questionnaire used to test the basic understanding of PD games.
- fig. S2. Interface for playing the PD game in anonymous treatment.
- fig. S3. Interface for playing the PD game in onymous treatment.
- fig. S4. Control trials.
- fig. S5. Regression diagnostics.
- fig. S6. Computer-simulated recreations of the anonymous treatment (T1).
- fig. S7. Computer-simulated recreations of the onymous treatment (T2).
- fig. S8. Comparison with other similar studies.
- table S1. Basic information on the experimental sessions.
- table S2. Gender as a confounding factor.
- table S3. Academic background as a confounding factor.
- Reference (*36*)

Download PDF

**Files in this Data Supplement:**

- Adobe PDF - 1601444\_SM.pdf
