# Supplementary material for: Onymity promotes cooperation in social dilemma experiments
Source: Sci Adv. 2017 Mar 29;3(3):e1601444. doi: 10.1126/sciadv.1601444 (PMC5371422; doi:10.1126/sciadv.1601444)
Supplement: http://advances.sciencemag.org/cgi/content/full/3/3/e1601444/DC1 [file 1601444_SM.pdf]

Science Advances

AAAS

advances.sciencemag.org/cgi/content/full/3/3/e1601444/DC1

Supplementary Materials for

Onymity promotes cooperation in social dilemma experiments

Zhen Wang, Marko Jusup, Rui-Wu Wang, Lei Shi, Yoh Iwasa, Yamir Moreno, Jürgen Kurths

Published 29 March 2017, *Sci. Adv.* **3**, e1601444 (2017)
DOI: 10.1126/sciadv.1601444

This PDF file includes:

- Supplementary Materials and Methods
- Supplementary Results
- fig. S1. Snapshot of the questionnaire used to test the basic understanding of PD games.
- fig. S2. Interface for playing the PD game in anonymous treatment.
- fig. S3. Interface for playing the PD game in onymous treatment.
- fig. S4. Control trials.
- fig. S5. Regression diagnostics.
- fig. S6. Computer-simulated recreations of the anonymous treatment (T1).
- fig. S7. Computer-simulated recreations of the onymous treatment (T2).
- fig. S8. Comparison with other similar studies.
- table S1. Basic information on the experimental sessions.
- table S2. Gender as a confounding factor.
- table S3. Academic background as a confounding factor.
- Reference (*36*)

Supplementary Materials and Methods

Experimental protocol and volunteer recruitment: The final decision on an experimental protocol for the present work was made in mid-June 2014. To ensure the comparability of our experiment with previous social dilemma experiments, we decided to adopt and--where necessary--minimally modify an existing experimental protocol (*22, 24*). We envisioned two treatments within this protocol: anonymous (T1) and onymous (T2). Each treatment consisted of multiple interactions and each interaction of multiple rounds, where *interaction* refers to a repeated Prisoner's Dilemma (PD) game between the same pair of opponents and *round* to a single repetition of that game. The original experimental protocol (*22, 24*) was closely followed in T1, but in T2, we needed to modify the protocol slightly to allow a pair of opponents to see each other's names. The described experiments were formally conducted from Sept. to Nov. 2014 over the course of three sessions (i.e., replicates) for each treatment. Experiments took place at the computer lab of Yunnan University of Finance and Economics, Kunming city, Yunnan province, south-western China. This lab was equipped with around 100 computer cubicles at the time, designed to minimize communication between participants during the experiment.

We recruited 154 undergraduate volunteers majoring in mathematics (55 persons), statistics (39), and eight other natural or social sciences (60), including foreign languages, environmental protection, public affairs, law, information technology, economics, media, and modern art. These volunteers came from three universities located in Kunming city: Yunnan University of Finance and Economics, Yunnan University, and Yunnan Agricultural University. To ensure anonymity in T1, in addition to keeping the current opponent's identity a secret, volunteers with different majors were chosen from different classes to the maximum extent possible. By contrast, to ensure meaningful onymity in T2, volunteers were chosen strictly from the same classes.

During the recruitment, no details on the experimental protocol were revealed to participants. Instead, participants were just required to show up at a designated location on the appointed date. The total of six sessions (three for each treatment) were carried out on Saturdays to avoid a possible conflict with scheduled lectures. Basic information on each session is shown in table S1. Important details pertaining to this table are as follows:

- The number of interactions was determined by two guidelines. First, participants were paired in a random order, but in such a way as to avoid having the same pair meet over and over again. If strictly observed, in a session with N participants, this guideline would have limited the number of interactions to N-1 (i.e., a given participant can only pair with N-1 other participants before having to interact with the same person again). Second, we planned each session to last about one hour and 15 minutes to obtain as much data as possible before participants started to lose concentration. Within this time frame, about 20 interactions could take place, which is reflected in the actual numbers of interactions reported in table S1.

- How many rounds would be played in a single interaction was determined randomly. Only the first round was certain, while the probability of extending the interaction by another round was set to 75%. To prevent participants from sitting idle, one random draw was made for all pairs, meaning that every participant played the same number of rounds. According to the described experimental setup, the expected number of rounds per interaction was 4 with an approximate standard deviation of 3.5. This setup, considered in conjunction with the actual numbers of interactions from table S1, is consistent with the total rounds per session reported in this table (approximately, 20 interactions×4 rounds per interaction=80 rounds).

- Because opportunities to recruit participants are limited, we tried to optimize the attendance in such a way that in about 20 anticipated interactions, every participant was matched up with everyone else without repetition (i.e., any given pair played only once). Consequently, the average attendance was 26.7 and 24.7 persons in T1 and T2, respectively.

- Within the student population from which we could recruit participants, the focus was on sophomores and juniors due to practical considerations. Namely, freshmen face the challenge of adapting to a new environment, while seniors are burdened with final exams. This choice was reflected in the corresponding values in table S1. Specifically, the average age of participants across all sessions was 21.6 years with a rather low standard deviation of 0.61 years.

- To avoid gender bias in the results, attempts were made to secure an equal number of female and male participants. Accordingly, 49.4% of all participants were women.

- Three persons in total were dismissed before even beginning the experiment because they failed to answer the questionnaire in fig. S1 correctly. This outcome is perhaps unsurprising given the simplicity of the said questionnaire. Nevertheless, because participants did answer our questions, we could be confident in their basic grasp of how the unilateral payoff matrix works, which is a minimum prerequisite to play the game meaningfully. Although a more complex questionnaire could be administered, doing so in future experiments would have to be weighed against a limited time window during which participants could be expected to stay fully concentrated.

Before the experiment could begin, each participant was randomly assigned to one isolated computer cubicle such that there was at least one empty cubicle between any two participants. Thereafter the experimental protocol was presented alongside instructions on how to play a repeated PD game. This presentation was followed by a simple questionnaire designed to test the basic understanding of the PD game (fig. S1). Only participants who answered the questionnaire correctly engaged in the formal experiment; others were given a show-up fee of ¥15 and subsequently dismissed. To avoid contaminating the results towards the end of each session, participants were kept unaware of the exact number of interactions. However, the probability of playing another round within a single interaction was made known. Participants also knew that they would be randomly rematched after the completion of every interaction. To prevent chatter, two supervisors were present at all times. If there was a problem, participants were allowed to call for help from supervisors by raising their hands.

Initially, all participants were assigned 50 start-up points. At the end of a session (each lasting around 1.25 h), participants converted their final score into a monetary gain. If the score had been negative, only the show-up fee of ¥15 was paid. Otherwise, the gain was calculated according to the exchange rate 1 pt=¥0.2, which yielded a range of gains from ¥15 to ¥31.6.

Experimental Platform and Interface: To allow participants to interact with one another, we built an experimental platform using the z-Tree software package (*34*). This platform contained two main interfaces which differed slightly between the two treatments (figs. S2, S3 display the interfaces for T1 and T2, respectively). In every round, participants were first shown the information needed to choose an appropriate strategy (figs. S2A, S3A) including:

- the number of the current round followed by the number of the current interaction in the top left box, e.g., 2/8 indicated the second round of the eighth interaction;

- the time remaining to make a choice in the top right box;

- a brief description in the middle box of the three possible strategic choices denoted by “1”, “2”, and “3” instead of *C*, *D*, and *P* in order to avoid biasing the results with positive and negative connotations of terms like “cooperation” and “defection”;

- the opponent's number (T1) or name (T2) and the current total payoff.

Strategic choices had to be made within 30 seconds by entering a number corresponding to the desired strategy (1, 2, or 3) into the light blue rectangle in figs. S2A, S3A. The choice was confirmed by clicking the “OK” button on the lower right side of the interface. For those participants who could not make a decision within the allotted time, a warning was prepared to appear on top of the screen reminding them to choose as soon as possible. However, in our experiment this warning has not been triggered. After all participants finished inputting their strategic choices, the system automatically moved to the second interface (figs. S2B, S3B).

By examining the second interface, participants could review the outcome of the current round. Compared to the first interface, only the bottom box changed. The newly displayed information included:

- own and opponent's strategic choices in the current round;

- own and opponent's payoff earned in the current round;

- the updated total payoff.

Similarly as above, this information could be reviewed for at most 30 seconds before a warning message was triggered. After all participants clicked the "continue" button in the lower right corner, the system switched back to the first interface to begin the next round or an entirely new interaction.

Upon the completion of an experimental session, participants were rewarded according to their final payoff and required to sign a receipt form to that effect. The experiment was approved by the Yunnan University of Finance and Economics Ethics Committee on the use of human participants in research and carried out in accordance with the approved guidelines.

Simulation methods: To gain deeper understanding of the underlying mechanisms responsible for generating the documented results, we recreate the experiment using computer simulations. In setting up the simulations, we use the simplest set of assumptions under which simulated behavior corresponds qualitatively, and even quantitatively, to the behavior of human participants (figs. S6, S7). Specifically,

- *First-order conditional strategies*. Having estimated the probabilities of possible responses to the opponent's action from the previous round (Fig. 2), we assume that simulated agents should, on average, exhibit the same behavior. More formally, let μ_{ij} denote the probabilities that the current action is $j \in \{C, D, P\}$ conditional on the opponent's previous action $i \in \{1^{st}, C, D, P\}$, where the element 1st signifies the first round, while *C*, *D*, and *P* respectively stand for cooperation, defection, and punishment. With such notation, μ_{ij} are precisely the probabilities given in Fig. 2. In the anonymous treatment (T1), for example, μ_{CD}=0.34.

- *Heterogeneity*. If agents were to blindly follow the first-order conditional strategies then simulated behaviors could not deviate much from the prescribed probabilities, μ_{ij}. In reality, however, each treatment produces almost the full spectrum of behaviors, ranging from unconditional defection to unconditional cooperation. Hence we assume heterogeneous agents, who follow the first-order

conditional strategies only on average. The corresponding mathematical formalism is that each agent has individualized probabilities of possible responses to the opponent's previous action, $x \in [0, 1]$, drawn from the exponentially distributed random variables, $p_{ij}=a \exp(bx)$, with a being the normalization constant and b chosen such that $\mathrm{E}p_{ij}=\mu_{ij}$, where E is the expectation operator. The exponential form is used solely for convenience.

- *Random mutation*. Strategies employed by individual agents do not have to stay constant over time. In particular, strategies can change on a whim (i.e., as a result of random "mutations") or rationally (i.e., through the process of cognitive selection). For simplicity's sake we assume the former, whereby a "mutation" event consists of a random pair of agents exchanging their strategies. In such a setup, agents can break the losing streak when their original strategy is maladjusted (e.g., a cooperator interacting predominantly with defectors). The mutation rates are small, 0.5% and 0.15% per interaction in anonymous (T1) and onymous (T2) treatments, respectively.

Supplementary Results

Control trials: In addition to anonymous (T1) and onymous (T2) treatments that represent the main interest of the present study, we organized two control trials, anonymous (C1) and onymous (C2), in which participants could not punish each other. Put alternatively, these control trials were the implementation of a traditional 2×2 PD game with *C* and *D* as the only possible strategies. The reason for performing these trials was to better understand our results relative to the previous similar studies (*22*, *24*). For example, comparing treatments T1 and T2 with control trials C1 and C2, would allow us to determine whether punishment improved cooperation as in Ref. *22* or failed to do so as in Ref. *24*. Moreover, if the behavior of participants changed between anonymous and onymous controls the same way it did between treatments T1 and T2, we would have further evidence in favor of the main conclusion of this study, i.e., that reducing onymity promotes cooperation in social dilemma experiments.

Control trails consisted of four separate sessions, two for the anonymous (C1) and two for the onymous (C2) control. All sessions were conducted following as much as possible the same experimental protocol as for treatments T1 and T2. The two anonymous sessions took place on 14 May 2015 (25 interactions, 84 rounds, 28 participants, mean age 21.9, standard deviation 0.79, 53.6% women) and 11 Nov. 2015 (21 interactions, 85 rounds, 32 participants, mean age 19.6, standard deviation 0.71, 65.6% women). The two onymous sessions were organized on 19 Sept. 2016 (24 interactions, 80 rounds, 30 participants, mean age 18.4, standard deviation 0.93, 73.3% women) and 20 Sept. 2016 (19 interactions, 83 rounds, 30 participants, mean age 18.7, standard deviation 1.08, 50.0% women). Overall, students who participated in the control trials were somewhat younger and covered a slightly wider age range than in treatments T1 and T2. Furthermore, women were better represented than men.

The results of control trials C1 and C2 qualitatively agreed with those of treatments T1 and T2 (fig. S4). In the anonymous control, C1, the frequency of defection reached a practically identical median value as in T1 and was much higher than the frequency of cooperation. Although the general pattern (i.e., a much higher frequency of defection than cooperation) was seen in both C1 and T1, the frequency of cooperation was significantly higher in the control trial (fig. S4A). We therefore concluded that the possibility to punish the opponent failed to increase cooperativeness. Our results upheld the conclusion of Ref. *24*. In fact, it would seem that when given the opportunity to punish, participants simply used this opportunity in place of cooperation without generating any prosocial value whatsoever. We also found that in C1 the payoff per round was negatively correlated with the frequency of cooperation (R^2=0.20, F=14.2, p=0.0004; fig. S4C). The same result was observed in T1, but only after accounting for the potential outliers (see Regression diagnostics below).

Much like the results of C1 agreed qualitatively with those of T1, the onymous control trial, C2, produced qualitatively the same results as T2. Quantitatively, however, onymity in C2 was even more successful in promoting cooperation than in T2. The median frequency of cooperation (defection) was significantly higher (lower) in C2 than T2 (fig. S4B), suggesting once again that punishment only stood in the way of cooperation. Furthermore, the payoff per round in C2, just as in T2, was positively correlated with the frequency of cooperation (R^2=0.565, F=75.4, $p<10^{-6}$; fig. S4D). Based on these results we were able to confirm that onymity is a powerful promoter of cooperation in social-dilemma experiments.

Comparing figs. S4D and 3D reveals that non-cooperative individuals earn a considerably higher payoff in control trial C2 than onymous treatment T2. This difference in payoffs is a direct consequence of punishment. Non-cooperative individuals get punished in T2, which negatively affects their payoff relative to C2. Cooperative individuals, by contrast, avoid punishment in T2 and thus maintain the same payoff level as in C2. A conclusion is that participants in our experiments use punishment against non-cooperative individuals, yet the desired prosocial effect of enticing cooperative behavior is nonexistent.

Gender as a confounding factor: To more completely understand the experimental results, we examined the role of gender as a confounding factor. In the anonymous treatment (T1), we had 39 female and 41 male participants. In the onymous treatment (T2), the number of both female and male participants equaled 37. How participants played (i.e., their strategic choices) depending on the treatment and gender is summarized in table S2. The results for both female and male participants exhibit a large variation across treatments. This variation is in line with the general pattern of the present study, meaning that both genders behaved more prosocially when anonymity was replaced with onymity. Beside this general pattern, the variation across genders is relatively small if a treatment is fixed, pointing to a similar behavior between female and male participants. This similarity may be due to a limited role of gender as a confounding factor. However, we also observe that in T1 female participants are somewhat less likely to cooperate (and more likely to defect) than their male counterparts, whereas the opposite is true in T2. To quantitatively examine which of these observations are more than a random occurrence, it is necessary to check for statistical significance.

Table S2 has the form of a three-way contingency table, implying that the relationship between the strategies played and the potential covariates (i.e., treatment and gender) can be disentangled by means of log-linear models. Specifically, we fit four log-linear model variants to the data to better understand the following issues:

1) To what extent does strategy vary across treatments irrespective of gender? For this question we resort to the model of joint independence consisting of the main effects (treatment, gender, and strategy) and treatment×strategy interaction.

2) Is an apparent effect of gender on strategy discernible after accounting for treatment? This question leads to the model of conditional independence that extends the joint-independence model with an extra (namely, treatment×gender) interaction.

3) Does the effect of gender on strategy vary across treatments? For this question we fit a homogeneous and a saturated log-linear model. The former model lacks the interactions between all three variables (treatment×gender×strategy) included in the latter model.

Amongst these four models, the goal is to select the simplest one that still fits the data relatively well. In this context, if the model of joint independence is selected, then the relationship between treatment and strategy is independent of gender. Selecting such a model would be sensible because we already know

that strategies vary considerably across treatments, but we cannot be sure if this variation is enough to fully explain the observed behaviors. Even if the joint-independence model fails to fit the data, we still lack a guarantee that there is a true effect of gender on strategy. Instead there may be an apparent effect that vanishes after accounting for treatment. Such a situation is indicated by the selection of the conditional-independence model. Finally, a true effect of gender on strategy may stay the same or may vary across treatments. The former (latter) is true if the homogeneous (saturated) model gets selected. These four models are nested. The joint-independence model is the simplest while the saturated model is the most complex. We perform the model selection using Bayesian information criterion (hereafter BIC); BIC can intuitively be thought of as a score awarding better goodness of fit, but penalizing lower degrees of freedom of more complex models (the lower the score, the better the model).

Upon fitting the four described models, BICs of joint-independence, conditional-independence, and homogeneous models were 22.7, 30.2, and 40.6, respectively, whereas BIC of the saturated model is 0 by definition. The saturated model got selected despite being the most complex alternative. These results point to a statistically significant effect of gender on the choice of strategy that varies between the treatments. In conjunction with table S2, we conclude that female participants were somewhat less likely to choose prosocial behavior than male participants when protected by the cloak of anonymity, yet they reverted to more prosocial behavior when this cloak was removed.

Academic background as a confounding factor: Another confounding factor for which we had sufficient data to analyze is the academic background. In the anonymous treatment (T1), a total of 80 students were evenly divided between mathematics and statistics (hereafter M&S) on the hand, and social sciences and humanities (hereafter SS&H) on the other hand. In the onymous treatment (T2), the number of M&S and SS&H students equaled 54 and 20, respectively. A summary of strategic choices depending on the treatment and academic background is given in table S3. The results are once again consistent with the general pattern of the present study, meaning that students with both backgrounds behaved more prosocially under onymity than anonymity. Unlike the results for gender, however, the variation across backgrounds was very small in T1, yet rather large in T2. Students with SS&H backgrounds were considerably more cooperative, less inclined to defect, and resorted to punishment only on a rare occasion after the veil of anonymity had been stripped.

For the purpose of a statistical analysis, we once more relied on log-linear models, i.e., the same four model variants already used in the analysis of gender as a confounding factor. Before performing the analysis, however, it was intuitively clear that the observations in table S3 fail to conform to any of the simplified interpretations offered by joint-independence, conditional-independence, and even homogeneous models. The reason behind such intuition is that, as described in the previous paragraph, behavior exhibited considerable variation not only across treatments (as in the case of gender), but also across backgrounds (in T2). Indeed, the statistical model selection was overwhelmingly in favor of the saturated model; BICs for joint-independence, conditional-independence, and homogeneous models were 1368, 684, and 348, respectively. We were forced to conclude that the effect of academic background on the strategies played varied across treatments. In particular, both groups of students behaved the same under anonymity and turned more prosocial under onymity, yet the change in behavior of the SS&H group was much more pronounced than that of the M&S group.

Regression diagnostics: To prevent the possibility of reaching erroneous conclusions based on regressions in Fig. 3 of the main text, we performed diagnostics aimed at identifying violations of the critical assumptions of the ordinary least square method. In particular, this method requires normally distributed residuals. If the distribution of residuals deviates from the normal distribution, there may be outliers with disproportionate effect on the estimated parameter values and the follow-up statistical inference.

We present the results of regression diagnostics in fig. S5. In five out of six instances the hypothesis of the residuals being normally distributed could not be rejected (fig. S5A). Only the case of cooperation in the anonymous treatment (shown in Fig. 3A of the main text) produced the residuals whose distribution deviated significantly from the normal one. Subsequent outlier detection identified four potentially problematic points. After removing these points the regression analysis was performed once again (fig. S5B). The results pointed to a weak negative correlation between the payoff per round and the use of cooperation in the anonymous treatment (R^2=0.06, F=4.71, p=0.033). Additional quantile regression on the full dataset confirmed this conclusion. In all other instances where the assumptions of the ordinary least squares method were upheld, the two types of regression (i.e., ordinary least squares and quantile) produced practically the same results.

Numerical simulations: To better understand what drives the observed behaviors, we recreated the experiment using computer simulations (see Simulation methods above). The simulation results agree qualitatively and quantitatively with the experimental outcomes of both anonymous (fig. S6) and onymous (fig. S7) treatments. To this end, we assumed only three straightforward mechanisms: (i) agents on average follow the first-order conditional strategies from Fig. 2, but (ii) individual behavior can deviate considerably from the probabilities prescribed therein, and (iii) agents change their strategies over time. Notably, assumption (iii) is implemented as random "mutations" to allow agents to discard maladjusted strategies. Such random mutations are in sharp contrast with a cognitive selection process that presumably takes place in real experiments due to the reasoning faculties of human participants. Nonetheless, the experiment and the simulations are in striking agreement, suggesting that cognitive selection effectively filters out maladjusted strategies, but may struggle to indicate well-adjusted ones. As for the "winners don't punish" effect, we find that the negative correlation between the payoff per round and the use of punishment persists for any linear combination of the first-order conditional strategies in Fig. 2. This effect, therefore, is strongly ingrained in the payoff structure of Eq. (1) and should be a consistently reproducible outcome of repeated PD experiments.

Comparison with similar studies: Although the present study was mainly preoccupied with the effects of onymity on cooperativeness, intriguing conclusions may be drawn by presenting our results in the context of other similar studies (fig. S8). In particular, Ref. *22* found that introducing punishment into a social dilemma experiment had a positive effect on the willingness to cooperate, albeit the most successful participants in this experiment--performed in Boston, Massachusetts--refrained from punishing others. An equivalent experiment--when implemented in Beijing, China (*24*)--failed to show the same beneficial effect of punishment. Because our treatment T1 is another reenactment of the same experiment, relating the findings herein to the findings of the aforementioned studies may shed a new light on the prosocial role of punishment. In doing the suggested comparison, it is important to keep in mind that the cultural differences between Boston and Beijing are bound to be much bigger than the differences between Beijing and Kunming (where the present study was executed). Nonetheless, Beijing and Kunming are separated by a distance of about 2000 km and regional distinctions within China may also be quite strong (36).

The results obtained in anonymous treatment T1 coincided closely with the results of the Beijing experiment (fig. S8). We did not find any statistically significant differences between the action frequencies in our T1 and those reported in Ref. *24*. Despite the lack of necessary data to conduct statistical inference on the Boston experiment, the average action frequencies from this experiment suggested a sharp contrast in how Chinese and American participants respond to the same social dilemma. Specifically, punishment appeared to be ineffective among Chinese participants. The overall conclusion was thus that our results, especially when interpreted in conjunction with the control trials, painted a bleak picture for the role of punishment in establishing cooperation, at least in a repeated PD game as a caricature of real-world social interactions.

调查问卷(Questionnaire)

在囚徒困境博弈中(见下面的收益矩阵)，你和你的对手同时进行策略选择。假如，你当前只有一个对手，你现在的总收益是 20，你和你的对手在这一轮分别选择策略“1”和“3”。那么，这轮你获得的纯收益是___-5___，这轮之后你的总收益是 ___15___。

假如你现在有两个对手，你现在的总收益仍是 20，你在这一轮选择策略“2”,你的两个对手分别选择为策略“1”和“3”，这轮你获得的纯收益是___-1___，这轮之后你的总收益是___19___。

收益矩阵(Payoff Matrix)

	你	对手
策略1	−1	+2
策略2	+1	−1
策略3	−1	−4

fig. S1. Snapshot of the questionnaire used to test the basic understanding of PD games. An English translation is as follows. Problem 1: In the PD game (see the accompanying payoff matrix), players make strategic choices simultaneously. Assuming that you have one opponent and your present total payoff is 20, if you and your opponent respectively choose strategies “1” and “3”, you earn ____ in the current round, and your total payoff becomes ____ after this round. Problem 2: Now you have two opponents at the same time. Your total payoff is still 20. If you choose strategy “2”, and your two opponents respectively choose strategies “1” and “3”, you earn ____ in the current round, and your total payoff becomes ____ after this round.

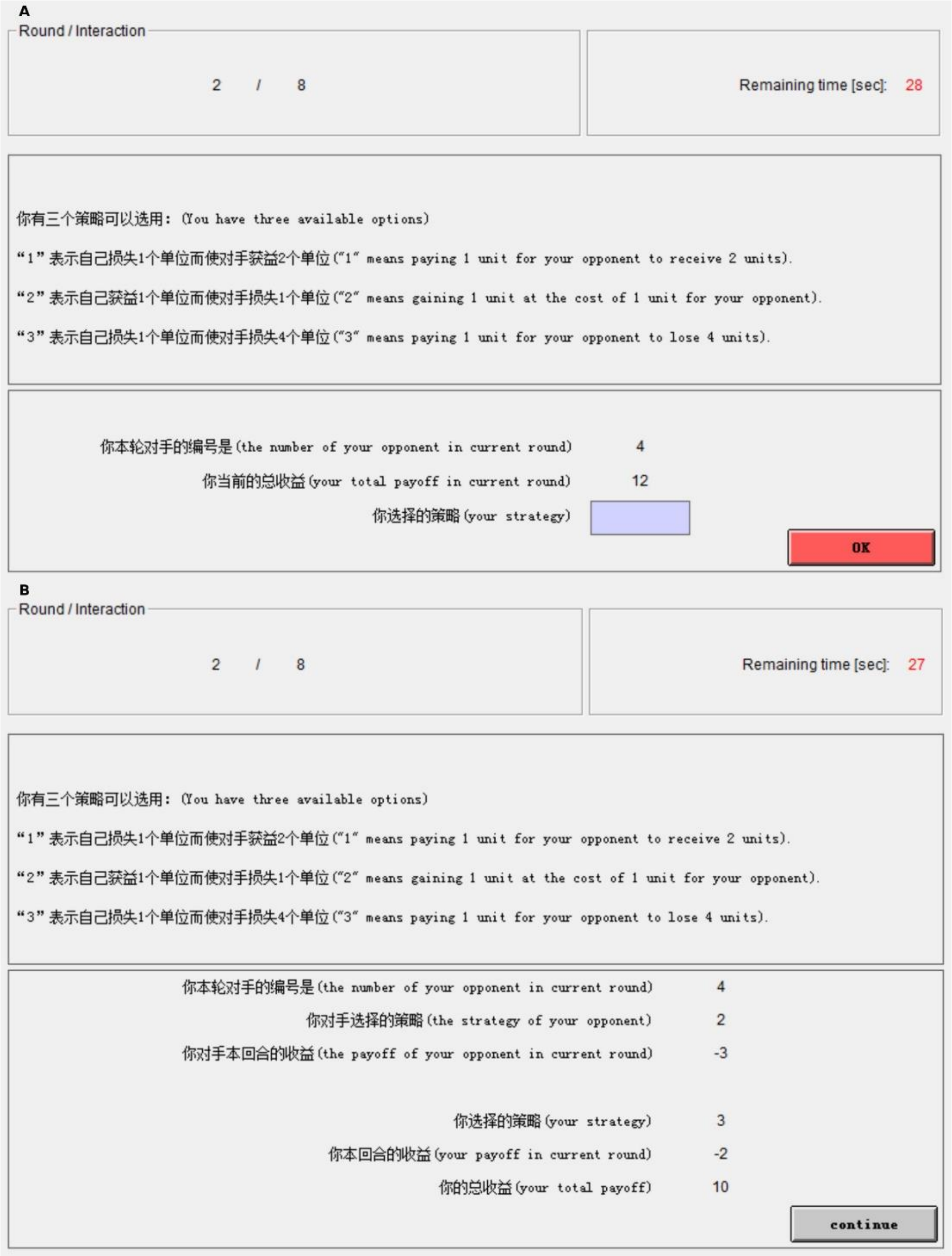

fig. S2. Interface for playing the PD game in anonymous treatment. A, Information available prior to making a strategic choice. **B**, Information available after the strategic choice was made. The opponent's identity was kept undisclosed.

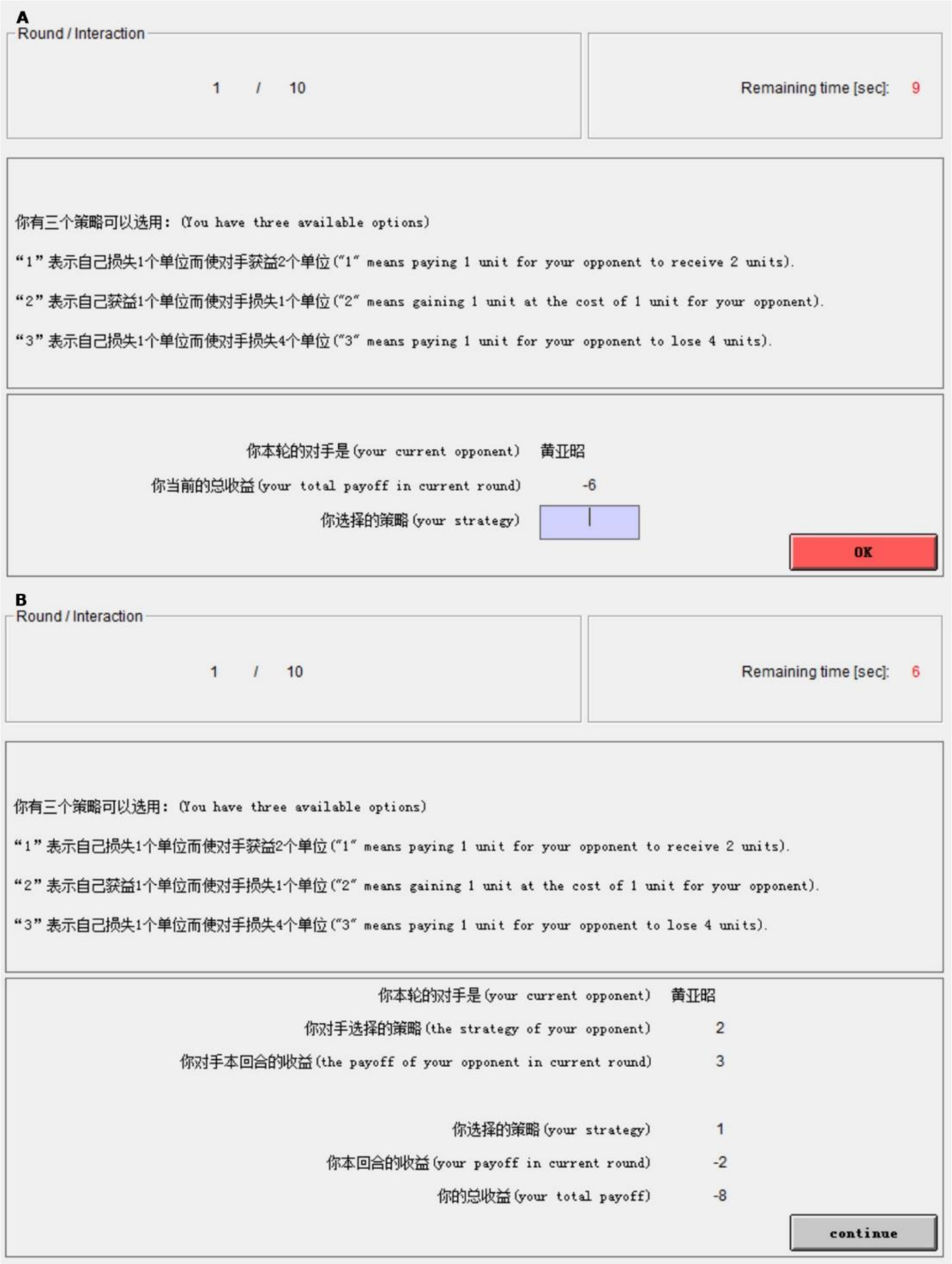

fig. S3. Interface for playing the PD game in onymous treatment. A, Information available prior to making a strategic choice. **B**, Information available after the strategic choice was made. Note that in this case the opponent's identity was disclosed.

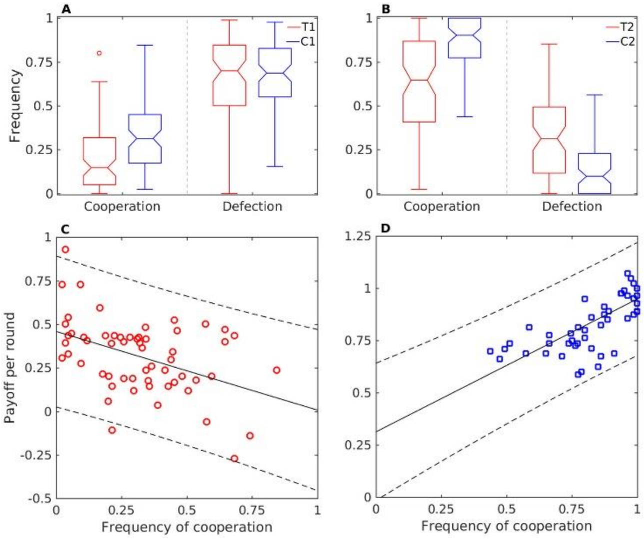

fig. S4. Control trials. In addition to the two main treatments, T1 and T2, an anonymous (C1) and an onymous (C2) control trial were organized to better relate this and similar studies (e.g., is punishment promoting cooperation or not; see *22*, *24*), and to strengthen the main conclusion of the present study (i.e., onymity promotes cooperation). **A**, The results in C1 were qualitatively similar to those in T1. Quantitatively, however, the frequency of cooperation was significantly higher in the control trial. **B**, Much like the relationship between T1 and C1, onymous control C2 was qualitatively similar to treatment T2, yet there were quantitative differences. In control trial C2, the frequency of cooperation (defection) was significantly higher (lower) than in T2. Overall, these results suggested that punishment not only failed to suppress defection, but also interfered with the willingness to cooperate. **C**, **D**, Correlations between the payoff per round and the frequency of cooperation were negative in C1 and positive in C2. These results agreed with those obtained in treatments T1 and T2, although in T1 we first had to account for the potential outliers.

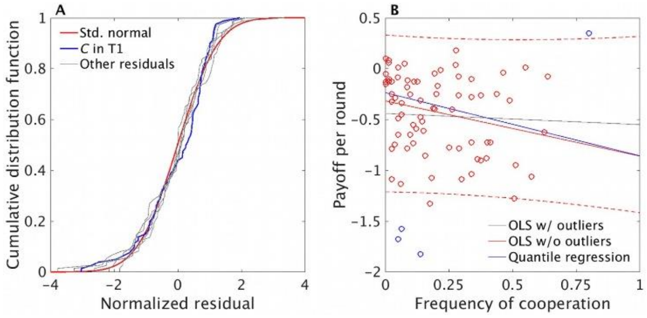

fig. S5. Regression diagnostics. A, Empirical cumulative distribution functions of the standardized residuals obtained by performing regressions in Fig. 3 of the main text are compared to the standard normal distribution. Generally the fit was good. Only in one out of six instances (*C* in T1; blue curve), the hypothesis of the residuals being normally distributed was rejected (Lilliefors test, D=0.132, p=0.0015). **B**, Outlier detection (*35*) identified four potential outliers in the dataset for *C* in T1 (blue dots). Redoing the regression analysis after the outliers had been removed suggested a weak negative correlation between the payoff per round and the frequency of cooperation (red line; intercept -0.318 with 95% confidence bounds -0.448 and -0.188; slope -0.542 with 95% confidence bounds -1.040 and -0.045). A similar result was obtained with quantile regression on the original dataset (blue line).

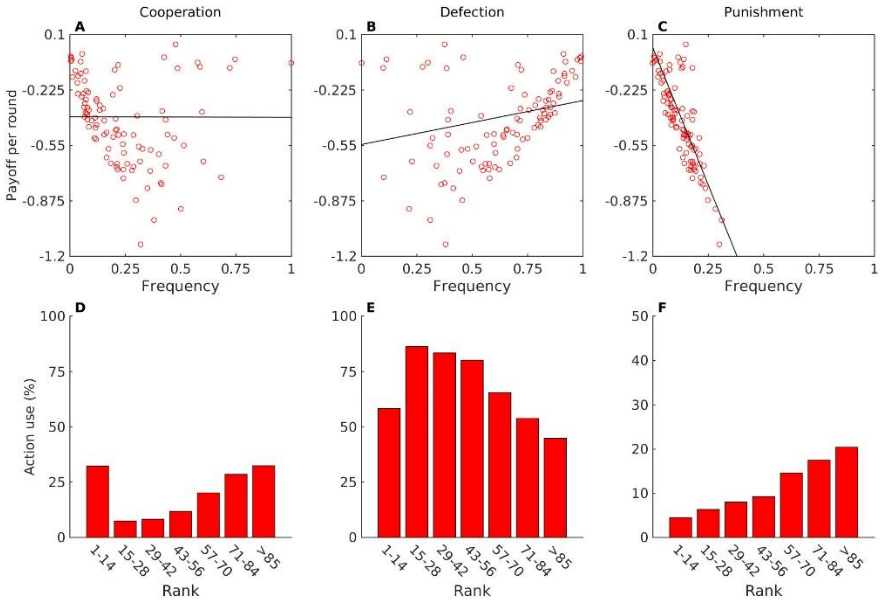

fig. S6. Computer-simulated recreations of the anonymous treatment (T1). The first-order conditional strategies (Fig. 2), along with heterogeneous and mutating agents, are sufficient to qualitatively and quantitatively recreate most of the observed experimental outcomes. **A--F**, After 100 interactions, the median payoff per round is -0.390. Individual payoffs per round are uncorrelated (weakly positively correlated) with the frequency of cooperation (defection). The mutation rate is 0.5% per interaction.

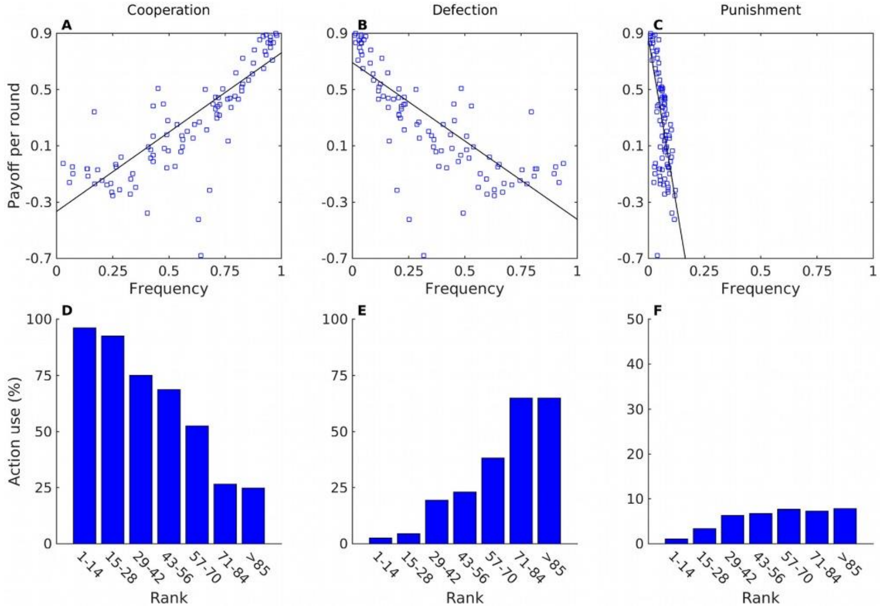

fig. S7. Computer-simulated recreations of the onymous treatment (T2). A--F, After 100 interactions, the median payoff per round is 0.306. Individual payoffs per round are positively (negatively) correlated with the frequency of cooperation (defection). The mutation rate is 0.15% per interaction. A lower mutation rate in T2 suggests that onymity offers a more reassuring environment in which actions are taken with firmer conviction. The "winners don't punish" effect is observable in both treatments.

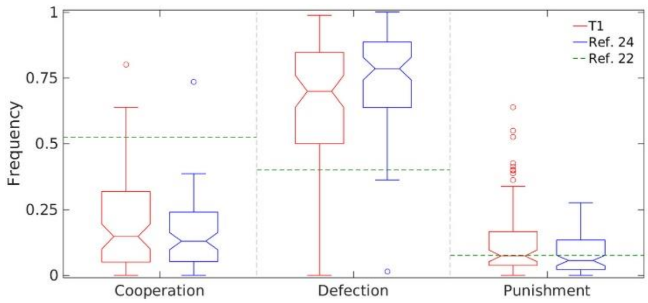

fig. S8. Comparison with other similar studies. The results of the present study in the anonymous treatment (T1) coincide closely with the results of an equivalent experiment performed in Beijing, China and reported in Ref. *24*. There are no statistically significant differences in the action frequencies between the two studies. In addition, an equivalent experiment was performed in Boston, Massachusetts and reported in Ref. *22*, but we lacked the necessary data to conduct statistical inference. Instead only the average action frequencies are shown (green dashed lines). A visual inspection suggests that there are considerable differences between how Chinese and American participants respond to the same social dilemma.

table S1. Basic information on the experimental sessions. A total of six sessions is divided equally between two treatments. Sessions are characterized by the number of interactions, the number of rounds, attendance, the mean age of participants and its standard deviation, and the percentage of women. The total numbers of dismissed participants who failed to answer the questionnaire from fig. S1 correctly is reported for each treatment.

Date	6 Sept. 2014	11 Oct. 2014	15 Nov. 2014	20 Sept. 2014	25 Oct. 2014	8 Nov. 2014
Treatment	T1			T2		
Interactions	21	19	20	23	22	20
Rounds	83	80	80	81	86	83
Participants	28	26	26	24	30	20
Mean age	21.5	21.9	21.4	22.9	21.5	21.4
SD age	0.51	0.65	0.50	0.59	0.63	0.50
% women	42.9	50.0	53.8	45.8	46.7	60.0
Dismissed	2			1		

table S2. Gender as a confounding factor. Frequencies in T1 were estimated using N_1^f=3159 and N_1^m=3321 data points for female and male participants, respectively. Frequencies in T2 were estimated using N_2^f=N_2^m=3082 data points for both female and male participants.

		C	*D*	*P*
Anonymous	Female	0.171	0.700	0.129
	Male	0.232	0.629	0.139
Onymous	Female	0.641	0.301	0.058
	Male	0.581	0.343	0.076

table S3. Academic background as a confounding factor. Frequencies in T1 were estimated using N_1^{ms}=N_1^{ssh}=3240 data points for both types of academic backgrounds. Frequencies in T2 were estimated using N_2^{ms}=4450 and N_2^{ssh}=1667 data points for mathematics and statistics (M&S), as well as social sciences and humanities (SS&H) backgrounds, respectively.

		C	*D*	*P*
Anonymous	M&S	0.201	0.659	0.140
	SS&H	0.204	0.668	0.128
Onymous	M&S	0.515	0.403	0.082
	SS&H	0.870	0.103	0.027
